# Neutral zone recording in computer‐guided implant prosthesis: A new digital neuromuscular approach

**DOI:** 10.1002/cre2.233

**Published:** 2019-08-16

**Authors:** Massimo Frascaria, Davide Pietropaoli, Matteo Casinelli, Ruggero Cattaneo, Eleonora Ortu, Annalisa Monaco

**Affiliations:** ^1^ Department of Life, Health and Environmental Sciences, San Salvatore Hospital University of L'Aquila L'Aquila Italy

**Keywords:** digital planning, implant, neutral zone, piezography

## Abstract

**Background:**

Neutral zone (NZ) is a specific area in the oral cavity where muscular opposite forces are null. NZ represents the ideal zone for prosthesis placement. In this study, we compared digital implant planning using conventional technique and using NZ registration through piezography.

**Methods:**

Sixty‐tree implants were digitally planned. Angular deviation differences between traditional planned and NZ‐planned implants were calculated. In addition, interferences with soft tissues (i.e., tongue and cheeks) were evaluated.

**Results:**

We observed a significant difference between traditional technique and piezographic approach in terms of implants angulation (*p* = .003), independent of site. A 4.7% of the planned abutments with traditional technique were placed outside the NZ, causing conflict with soft tissues in the digital model.

**Conclusions:**

Compared with traditional technique, piezography allows a significantly different exploitation of the nonconflict area, which potentially translates into better management of soft tissues and improved functionality of the implants.

## 1 INTRODUCTION

Recently, the increasing demand for full‐arch rehabilitations supported by dental implants imposes to ponder over the physiological integration of the prosthesis. In order to obtain patient satisfaction, the adequacy of both function and esthetics remains a key factor (Boven, Raghoebar, Vissink, & Meijer, 2015; Drago & Howell, 2012). The achievement of functional integration is probably the most challenging goal to attain for the clinician (Bonnet et al., 2016; Montero, Castillo‐Oyagüe, Lynch, Albaladejo, & Castaño, 2013). As widely reported by scientific literature, in fact, the prosthesis integration depends on static and dynamic factors, including soft and hard tissues that constitute the denture borders and forces among them (Laveau, 1986; Miller, Monteith, & Heath, 1998; Sutton, Glenny, & McCord, 2005). According to this, prosthetic volume should be placed in a nonconflict area defined as neutral zone (Fahmi, 1992; Fahmy & Kharat, 1990; Ohkubo, Hanatani, Hosoi, & Mizuno, 2000; Porwal & Sasaki, 2013), a specific space in which the opposite muscular forces are equal to 0 (Cagna, Massad, & Schiesser, 2009; Porwal & Sasaki, 2013). This assumption is corroborated by the evidence that natural teeth tend to fill the NZ space between tongue, lip, and cheek according to muscles effects and occlusal contact (Cagna et al., 2009; Moss, 1997; Okeson, 2014). Teeth volume, angulation, and diameter, in fact, are not accidental, and the denture should be manufactured according to these physiological parameters (Cagna et al., 2009). NZ area can be recorded using an impression technique named piezography, which is a registration of the space defined by muscles pressure between tongue, lip, and cheek during functional activity (e.g., speak and swallow; Ikebe, Okuno, & Nokubi, 2006; Klein, 1974, 1989).

When an implant‐supported rehabilitation is planned, proper placement of fixtures is a keystone for its long‐term stability and predictability, considering that implant position cannot be modified after surgery Esposito, Maghaireh, Grusovin, Ziounas, & Worthington, 2012. The correct implant placement and an ideal prosthetic design affect also the stability of implant components, involving perfect fitting between implant connection system and prosthetic structures (Bramanti et al., 2017; Cicciù et al., 2014; Cicciù et al., 2015). The predictability of the fixture placement can be enhanced using current technologies, namely, computer‐aided design (CAD) and computer‐aided manufacturing (CAM) during pre‐surgical planning Barone, Casinelli, Frascaria, Paoli, & Razionale, 2014. CAD/CAM workflow allows the clinician to combine multiple data before surgery, such as tridimensional (3D) radiographies, anatomical structures data, intraoral and extraoral volume scans, mandible movements, and additional information that can enhance rehabilitation quality (D'haese, Ackhurst, Wismeijer, De Bruyn, & Tahmaseb, 2017; Joda, Ferrari, Gallucci, Wittneben, & Brägger, 2017). This approach provides a virtual patient‐personalized environment in which the denture can be digitally designed and tested before the prosthetic procedures, promoting their physiological integration (Barone et al., 2014; Frascaria et al., 2015). Often, however, fixtures are placed in order to primarily respect residual bone crest and rehabilitation esthetics (Benic, Elmasry, & Hämmerle, 2015; Dolcini, Colombo, & Mangano, 2016), with marginal attention to the structures recorded by piezography. The magnitude of this oversight is often underestimated during the surgical planification, but it comes tremendously severe after denture placement, causing annoying dilemmas for both clinicians and patients such as biting of tongue, lip, or cheeks, patient discomfort (Devlin & Hoad‐Reddick, 2001; McCord, 2009), or problems with fitting between the prosthetic components and resulting stability of biomechanical systems (Cervino et al., 2018; Cicciu, Bramanti, Matacena, Guglielmino, & Risitano, 2014; Cicciù, Risitano, Maiorana, & Franceschini, 2009; Lauritano et al., 2016). Interestingly, a recent case report described the fabrication of full‐arch CAD/CAM rehabilitation using NZ data in order to mold the denture volume in the nonconflict area, thus increasing its stability as well as patient satisfaction and achieving a more physiologically integrated rehabilitation (Ohkubo, Shimpo, Tokue, Park, & Kim, 2017).

To our best knowledge, no studies have investigated how piezography can influence implant placement during CAD/CAM workflow. From this basis, our aim was to compare the traditional versus the neuromuscular (piezographic) digital implant planning approach in terms of invasion of the NZ area.

## 2 MATERIALS AND METHOD

We conducted a descriptive pilot study comparing two digital strategies of presurgical planning of fixed prosthesis in patients requiring implants among those who accessed the dental clinic of the University of L'Aquila (Italy) from September 2014 to June 2017.

Our research fulfilled the principles stated in the Declaration of Helsinki (Declaration of Helsinki, n.d.) and obtained approval from the local Institutional Review Board.

Patients who fulfilled the following characteristics were eligible for the study: males and females aged at least 25 years, implant‐sustained prosthetic demand, partial edentulism of class III or IV according to the American College of Prosthodontists (McGarry et al., 2002), and acquired informed consent. Subjects were excluded if any of the following criteria were met: systemic disease that would interfere with dental implant therapy; any contraindications for oral surgical procedures; any contraindications for radiodiagnostic procedures; smoking habits >10 cigarettes per day or equivalents‐chewed tobacco; physical or mental handicaps that would interfere with the ability to perform adequate study protocol; previous implant rehabilitation; inadequate oral hygiene or unmotivation for adequate home care; pregnant; or breastfeeding women (Pietropaoli et al., 2013).

### 2.1 Digital planning strategies

#### 2.1.1 Traditional digital workflow

**Figure 1:**
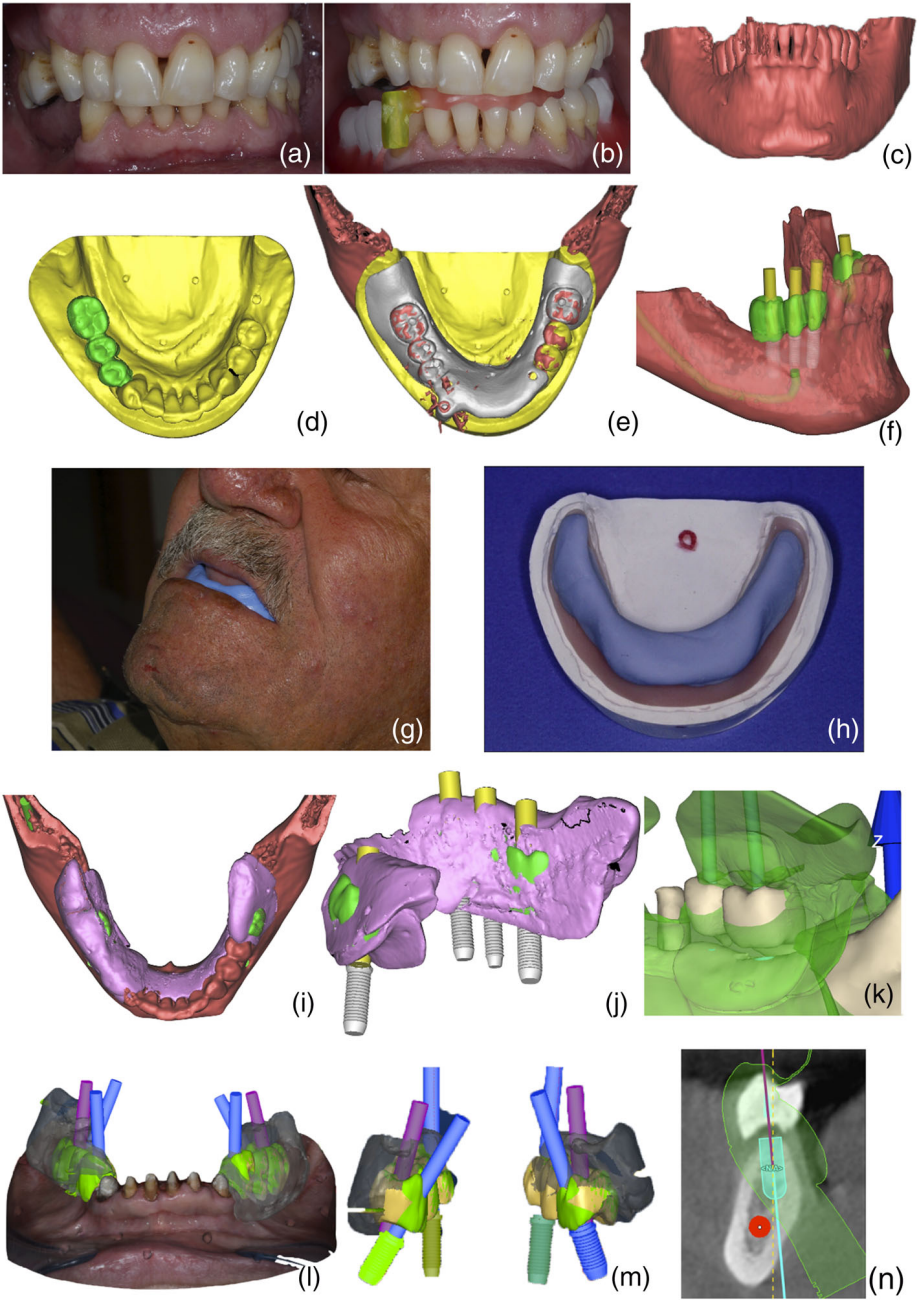
Study procedures were summarized in those panels. (a) Patient involved in the study. The tongue overrun edentulous space. (b) Radiographic guide check on edentulous arch. (c) Cone beam–computed tomography 3D rendering. (d) Optical scan data. (e) Integration of surface anatomical data, from surface scanner, and 3D radiographic data. (f) Computer and prosthetic guided implant planning. (g) Neutral zone (NZ) registration procedures. (h) Piezographic record on its model. (i) NZ registration integrated in the virtual environment using the model cast as reference. (j) Implant planning based on piezographic data. (k) Traditional teeth arrangement does not respect NZ on the lingual side. (l,m) Differences of implant axes planned on traditional teeth arrangement technique (in blue) and on piezographic data (in violet). (n) Piezography‐based implant planning in cross‐sectional view. The software generates angular measurement referring to the dotted line, which is perpendicular to occlusal plane. Note the invasion of conflict zone by tooth of radiographic template

In order to plan CAD/CAM implants, the enrolled subject underwent impressions for dental casts, occlusal jaws recordings, and cone beam–computed tomography (CBCT) as required by standard digital workflow (Flügge, Nelson, Schmelzeisen, & Metzger, 2013; Frascaria et al., 2015; Vercruyssen, Laleman, Jacobs, & Quirynen, 2015). Briefly, a wax‐up was produced for each patient in order to previsualize final restoration; then, a diagnostic radiopaque template was generated from the wax‐up and placed directly over the edentulous ridge in the required occlusion condition, according to traditional prosthetic techniques (Figure 1a). The patients underwent CBCT analysis wearing the radiopaque diagnostic prosthesis (Figure 1b,c), and the dental casts were then digitized together with the same template using specific 3D scanner (Figure 1d,e).

Virtual dental casts, jaws recordings, and CBCT data were acquired by a specific digital implant planification software (NemoScan, Nemotec, Madrid, Spain). Bone structure (CBCT data) and dentition models (optical scan data) were digitally integrated through identification of at least three common points (anatomical areas and/or radiographic templates).

#### 2.1.2 Piezography‐incorporating technique

A custom‐made trial denture base was produced and placed maxillary. Registration of NZ space was carried out by piezography using acrylic impression resin (Bosworth Sapphire, The Bosworth Company, Skokie, IL, USA). Briefly, acrylic resin was prepared with a ratio of two parts of powder and 0.8 part of liquid, mixed up to obtain gummy‐like compound, and then placed maxillary onto the trial after teeth lubrication for preventing adhesion (liquid vaseline). As reported by several authors (Alfano & Leupold, 2001; Beresin & Schiesser, 2006; Fahmy & Kharat, 1990; Klein, 1974, 1989; Makzoumé, 2004; Porwal & Sasaki, 2013), patients were instructed to repeat phonetic sounds (e.g., “sis,” “se,” “so,” “te,” “de,” “moo,” and “sees”) and invited to swallow, laugh, and kiss, moving lips and cheeks in order to mold the core of the piezographic space. These phases were repeated at least three times. At the end of the piezographic procedure, the molded resin was adapted on the maxillary cast for completing its drying. Piezography with dental cast was then scanned, producing a virtual model. NZ registration phases are described in Figure 1g,h. NZ registration was then integrated in the virtual environment using the model cast as reference (Figure 1i).

### 2.2 Evaluation of oral‐buccal angulation of fixtures: Traditional digital workflow versus piezography‐incorporating technique

Ideal fixtures placement should include only compressive axial loading, and the implant vertical axis should fall in the middle of the perimeter identified by the cuspidal crests of the tooth occlusal plane (Barone et al., 2014). From this assumption, digital workflow was first performed with traditional technique and then replicated using piezography‐based approach, in order to verify comparability of the two methods and whether traditional technique respects the NZ.

According to the traditional technique (Barone et al., 2014; Frascaria et al., 2015; Vercruyssen et al., 2015), namely, considering the residual alveolar bone crest and crown emergency on gums, occlusal contacts, and esthetics, a single clinician expert on digital surgery procedures (M. F.) planned the position and size of fixtures–abutment components (Figure 1f). Using the same software, the oral‐buccal angulation of fixtures was calculated, setting the occlusion line as a reference.

Afterward, the same operator acquired patients piezography scan and reallocated it onto the previously obtained planification model using a specific software, to verify the anatomical relationships of the model with NZ (Figure 1j,k). Subsequently, the operator proceeded to a new planification that took into consideration the virtual space represented by the NZ area as delimited by piezography. As before, fixtures angulation was measured so that NZ was not invaded after implants placement (Figure 1l–n).

### 2.3 Statistics

Data relative to fixtures angulation and location of receiving site were recorded in a spreadsheet. Absolute difference in angulation between fixtures placement with and without piezography was calculated and used for differential statistics. Data normality was verified by Shapiro–Wilk test. Paired *t* test was used for testing difference between groups. Statistical significance was set at *p* < .05. Graphs and statistics were generated by automated functions written in R (Team, 2014) using a public domain libraries “ggplot2” (Moon, 2016) and “stat” (Team, 2014), respectively. Graphs report mean and standard error (SE), unless otherwise specified.

## 3 RESULTS

Fourteen subjects (eight males/six females) who needed implant placement were recruited, for a total of 63 digitally planned implants. Each implant was planned using both standard and piezographic approach.

**Figure 2:**
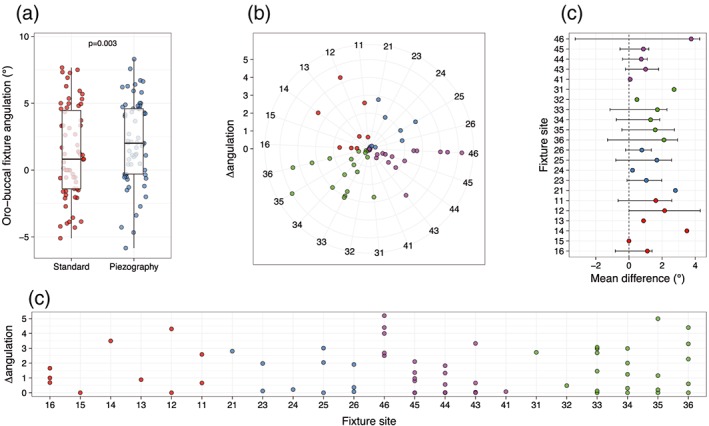
(a) Boxplot of mean differences in terms of angulation between standard and piezographic implant placing. (b) Polar plot of placed implant. Axis expresses implant sites, whereas the dots represent single implants. Distance between the centers of the circle indicates mean differences between the two techniques. (c,d) Mean differences were reported in both graphs

We observed a significant difference between traditional technique and piezography‐based approach in the mean oral‐buccal angulation of digitally planned fixtures (1.30; 95% CI [0.44, 2.17]; and 2.08; 95% CI [1.29, 2.87], respectively; mean difference: −0.78; 95% CI [−1.28, −0.28], *p* = .003). Moreover, piezography‐incorporating technique resulted in a better exploitation of the nonconflict area compared with traditional planning. In fact, although 100% of fixtures that had been digitally placed by piezographic approach respected the NZ, 4.7% (*n* = 3) of those planned with the traditional technique were placed outside the NZ, thus causing severe conflict with soft tissues in the digital model (Figure 2). The between‐technique difference in angulation of digitally planned fixtures was not affected by the site (arch, dental element, or both; arch: Pearson's product‐moment correlation *r* = 0.02672; 95% CI [−0.2225, 0.2727], *p* = .8353; dental element: *r* = 0.1055; 95% CI [−0.146, 0.3443], *p* = .4103; both: *r* = 0.04072, 95% CI [−0.2092, 0.2856], *p* = .7514).

**Figure 3:**
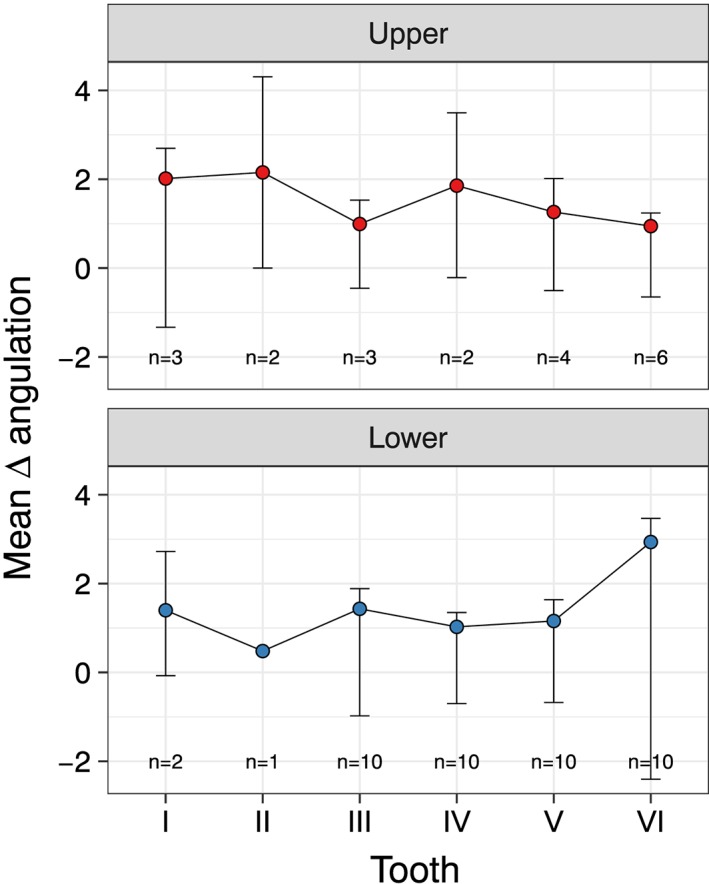
Analysis of mean differences stratified per arch. *N* represents the number of implant placed on tooth side

Even without reaching statistical significance, the greatest difference between traditional technique and piezographic planification was observed at the lower arch (Figure 3). In fact, the mean delta angulation was 1.60 ± 1.56 at the lower arch and 1.39 ± 1.32 at the upper arch. No differences were observed among quadrants, sextants, or elements in terms of delta angulation.

## 4 DISCUSSION

The results of our pilot study on 63 implants suggest that NZ significantly influences the positioning of implant fixtures. Compared with traditional technique, in fact, piezography‐incorporating approach allows a significantly different exploitation of the nonconflict area, which potentially translates into better management of soft tissues and improved functionality of the implants. Indeed, implant fixture placement could be facilitated and improved by means of a prosthetic guided approach, in which the surgical planning and the prosthetic manufacturing rely on biomechanical, functional, and esthetic requirements of a specific patient (Barone et al., 2014; Frascaria et al., 2015).

Most of the traditional prosthetic techniques only consider static evaluations, such as arrangement of posterior denture teeth directly over the crest of the edentulous ridge (Cagna et al., 2009). As such, they ignore the impact of neuromuscular activity, which develops during childhood and then changes throughout life, on rehabilitation outcomes. Dynamic functions, indeed, remain highly individual also in the edentulous patient and influence the performance of any rehabilitation device placed in the mouth (Beresin & Schiesser, 2006). NZ detection techniques are widely used in removable denture manufacturing protocols, in order to avoid NZ invasion and to improve prosthesis stability, as well as phonation and soft tissue support accordingly (Beresin & Schiesser, 2006; Cagna et al., 2009). Conversely, there is no information about the effect of techniques incorporating NZ measurement on fixed implant‐supported prosthesis outcome, neither in terms of functional adaptation nor about implant stability. In our pilot study, we observed for the first time that angular deviation of fixture planning axis provides accurate quantification of the amount of NZ invasion by traditional prosthetic design (Figure 1i–k and 1n).

The functionality of a dental prosthesis with respect to biomechanical and esthetic characteristics requires a well‐designed implant positioning (Barone et al., 2014). The prosthetic design, in fact, is substantially determined by the implant position, and this cannot be changed after surgery. In particular, implant axes affect crown profiles emergence, as well as occlusion dynamics and oro‐lingual position. Clinicians may resort to prosthetic compensation in case of suboptimal fixture placement, but this solution is not always effective for a good outcome of rehabilitation when the fixtures are misplaced. Theoretically, clinicians should detect the ideal individual prosthetic volume before implant surgery in order to avoid mistakes in fixtures placement. In this sense, the use of 3D imaging and planning softwares may improve the surgical strategy through the integration of different diagnostic parameters, thus allowing a comprehensive approach to the question (Frascaria et al., 2015). Here, we demonstrate that the entire presurgical planning process can be carried out using CAD applications that combine different maxillofacial diagnostic modalities: radiographic data, captured by a CBCT scanner, and surface anatomical data, acquired by a structured light scanner.

The described approach offers several advantages. First, it personalizes the analysis of the prosthetic space with regard to fixtures positioning. Second, the accurate preliminary assessment of both hard and soft peri‐implantar tissues allows the precise determination of the available prosthetic space and the choice of the appropriate implant system and prosthetic components. In turn, an accurate reconstruction of oral surfaces improves outcome predictability following implant placement (Frisardi et al., 2011). An additional advantage of piezography in digital planning is that of relying on objective parameters rather than subjective visual perceptions during the reconstruction process. Most importantly, the proposed approach can particularly assist the clinician in the rehabilitation of the edentulous patients. After natural teeth loss, in fact, the potential denture space lies within the mouth between the tongue thrust, pressing outward, and the forces of the cheeks and lips, pressing inward (Beresin & Schiesser, 2006). The potential denture space in long‐time edentulous arches is therefore different from dentate condition, as a consequence of muscular activity adaptation Monaco, Sgolastra, Pietropaoli, Giannoni, & Cattaneo, 2013. Thus, the prosthetic teeth should be placed on edentulous arches according to individual neuromuscular function (Chipaila et al., 2014).

In conclusion, piezography appears as an effective additional technique in customized implant planning and implant‐supported prosthesis manufacturing, and the piezography‐incorporating approach allows a significantly different management of the NZ compared with traditional digital planning. Further studies are needed to test the short‐ and long‐term clinical effects of the neuromuscular approach on functionality and durability of implants and on their reciprocal relationship with soft tissues and dynamic forces.

## DISCLAIMERS

None.

## FUNDING INFORMATION

None.

## CONFLICT OF INTEREST

None.
